# Expression of the RPSA-Containing and 67EBP Laminin Receptors in Relation to the Debatable Nature of the 67 kDa Laminin Receptor 67LR in Colorectal Cancer

**DOI:** 10.3390/ijms26062564

**Published:** 2025-03-12

**Authors:** Gabriel Cloutier, Taoufik Khalfaoui, Julie C. Carrier, Jean-François Beaulieu

**Affiliations:** 1 Laboratory of Intestinal Physiopathology, Department of Immunology and Cell Biology, Faculty of Medicine and Health Sciences, Université de Sherbrooke, Sherbrooke, QC J1H 5N4, Canada; 2 Department of Medicine, Faculty of Medicine and Health Sciences, Université de Sherbrooke, Sherbrooke, QC J1H 5N4, Canada; 3 Centre de Recherche du Centre Hospitalier Universitaire de Sherbrooke, Sherbrooke, QC J1H 5N4, Canada

**Keywords:** colorectal cancer, laminin receptor, elastin-binding protein

## Abstract

The role of laminin receptors in colorectal cancer (CRC) is the subject of ongoing research. Histopathological studies have suggested that the 67 kDa laminin receptor (67LR) is involved in the carcinogenesis of various malignancies, including CRC. However, the exact composition and nature of 67LR have been a source of confusion for many years. A recent study from our group reported that the 37 kDa form of RPSA participates as a laminin receptor renamed the RPSA-containing laminin receptor (RCLR) but is not the precursor form of the 67LR since the 67 kDa protein associated with 67LR corresponds to the 67 kDa elastin-binding protein (67EBP), which also acts as a laminin receptor. The present study aims to analyze the distinct expression patterns of these two laminin receptor components in CRC. Expressions of RCLR and 67EBP were analyzed in CRC tissues using Western blot and quantitative RT-PCR analyses. The primary colorectal adenocarcinoma tissues and corresponding resection margins showed an overexpression of both RPSA and 67EBP at the protein level in the CRC tissues. An analysis of the publicly available CRC datasets confirmed the overexpression of RPSA and 67EBP in CRC tissues. In conclusion, the elevated expression of these two non-integrin laminin receptors in CRC lesions suggests their critical roles in colorectal carcinogenesis and emphasizes their potential usefulness as tissue biomarkers.

## 1. Introduction

Colorectal cancer (CRC) is a significant public health issue and remains one of the leading causes of cancer-related deaths. The development and progression of CRC are complex processes influenced by various factors, including interactions between cancer cells and the basement membrane (BM). The intestinal BM is a specialized extracellular matrix separating the epithelial and connective tissues and is composed of several glycoproteins such as laminins, collagens, proteoglycans, fibronectins, and fibrin [1,2]. Laminins located at the epithelial–stromal interface have been shown to be central to the regulation of intestinal epithelial cell proliferation, migration, survival, and differentiation in both healthy and pathological conditions through interactions with specific receptors of the integrin and non-integrin families [3,4,5,6,7]. Some of these receptors have been shown to regulate normal and CRC cell proliferation, such as α7β1 and the 67 kDa laminin receptor 67LR [8,9,10]; intestinal cell adhesion and migration, such as 67LR [8,10,11,12]; or cell differentiation, such as α6Bβ4, while α6Aβ4 has been identified as a pro-proliferative integrin in colorectal cancer cells [6].

The 67LR was the first laminin receptor to be described [13,14]. In the years following its discovery, the 67LR was reported to be overexpressed in neoplastic cells, contributing to aggressiveness and poor prognosis in a wide variety of cancers [15], including CRC in which the 67LR expression has been correlated with disease progression [16,17,18,19]. As mentioned above, a number of studies have provided data supporting that adhesion, migration, and proliferation can be mediated by the 67LR in CRC cell lines. However, the mechanisms by which 67LR regulates these cell functions remain poorly understood due to limited knowledge of the receptor’s biochemistry and biology.

Indeed, following the 67LR isolation, antibodies developed against the purified receptor from a laminin affinity column allowed the recognition of a peptide corresponding to a sequence present on the 37 kDa ribosomal protein SA (RPSA) [18,20,21]. Thenceforth, it has been commonly accepted that the RPSA protein can act as a precursor to the 67LR, although the mechanism by which the RPSA protein can become a 67 kDa membrane receptor for laminin remains elusive [22,23,24]. Incidentally, post-translational modifications, followed by covalent homodimerization or heterodimerization of RPSA with another unidentified partner, were among the proposed hypotheses for the mature 67LR formation [23,25,26], but the absence of identification of a dimerization partner left doubt surrounding the exact identity of the mature receptor for many years [24].

However, a recent study from our group using cell fractionation of CRC cells pointed out that the 67 kDa immunoreactive protein, corresponding to the mature 67LR, was present in the soluble protein fraction and identified as another laminin receptor displaying immune cross-reactivity with RPSA, the β-galactosidase-related 67 kDa elastin-binding protein (67EBP) [27]. Furthermore, a small proportion of the 37 kDa RPSA exhibited membrane-like properties [27]. Considering these new insights that the 67 kDa immunoreactive protein, historically associated with 67LR, corresponds to 67EBP, and the clarification that the 37 kDa RPSA can act as a RPSA-containing laminin receptor (RCLR) [27], the expression of these two receptors needs to be revisited in the context of CRC. Our working hypothesis was that both of these laminin receptors could be involved in CRC.

## 2. Results

### 2.1. RPSA and 67EBP Are Overexpressed at the Protein Level in CRC Tissues

**Figure 1:**
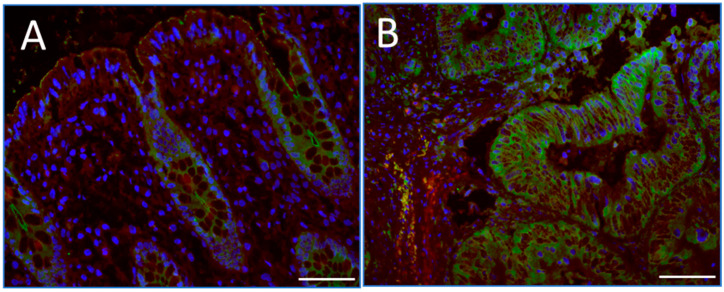
Immunodetection of immunoreactive RPSA in CRC tissues. Representative images of indirect immunofluorescence staining using an anti-RPSA antibody on a human CRC stage III sample and its matched adjacent normal mucosa. The staining was weak and restricted to the proliferative area of the resection margin, (**A**) while it was more intense and widespread in carcinoma (**B**). Some of the staining can be attributed to the presence of 67EBP considering the sequence homology with RPSA. Scale bars = 50 μm.

The 67LR has frequently been reported to be overexpressed in several cancers including CRC as mentioned above by immunohistochemical methods using antibodies that cross-react with a 67 kDa antigen [18,19]. The expression of RPSA in colorectal tumors and normal mucosa corresponding to the resection margins was evaluated by indirect immunofluorescence on cryosections using an anti-RPSA antibody. Using this antibody, we detected relatively weak staining located in the crypts in normal intestinal mucosa, where proliferative epithelial cells are located, as expected from our previous study [8], compared with relatively intense staining throughout parenchymatous cells in carcinoma tissues (Figure 1). This observation may be indicative of higher levels of RPSA in CRC although an increase in 67EBP cannot be excluded considering the cross-reactivity of the anti-RPSA antibodies with 67EBP previously observed and the identification of putative sequences responsible for the cross-reactivity [27].

**Figure 2:**
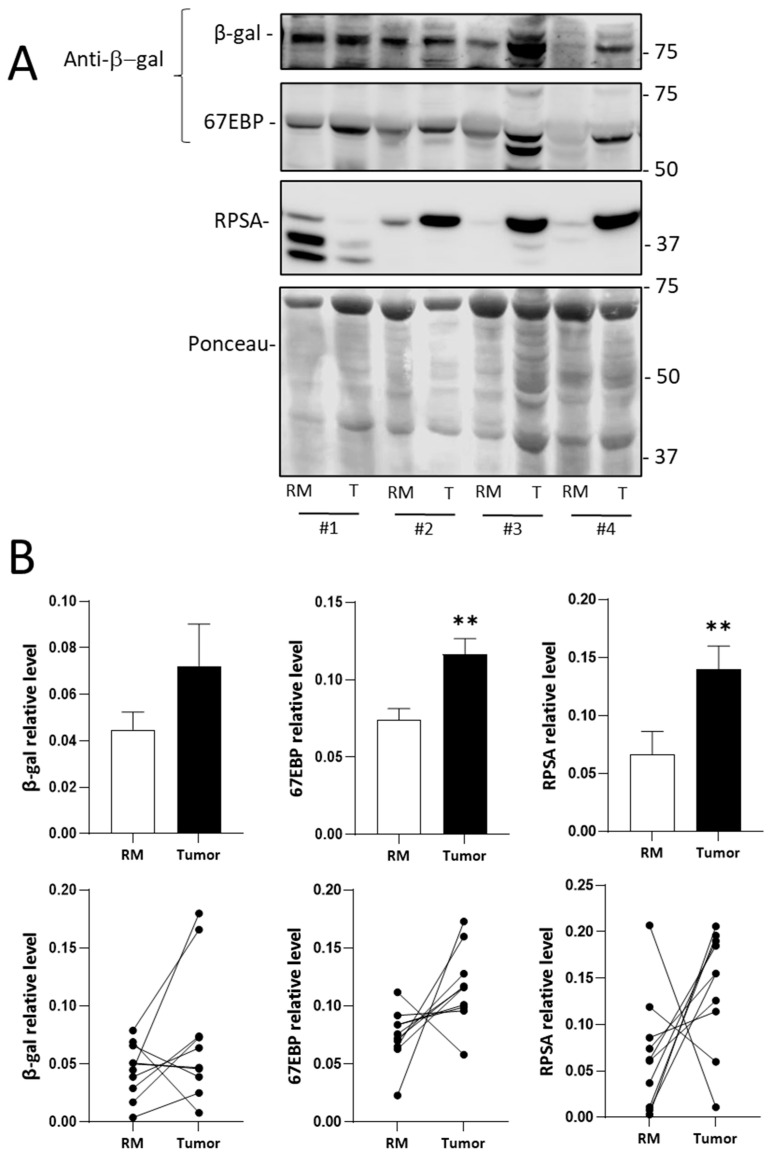
Immunodetection of RPSA, 67EBP, and β-gal proteins in CRC tissues. RPSA and 67EBP are overexpressed at the protein level in CRC tissues. (**A**) Representative Western blots showing the detection of immunoreactive proteins corresponding to β-gal, RPSA, and 67EBP in four representative colorectal tumors and their resection margins (labeled #1 to #4) from CRC sample collections (Series #1, Table 1). Ponceau red staining was used as the protein loading control. (**B**) Graphs displaying the relative amounts of β-gal, RPSA, and 67EBP as determined by densitometry analysis using Ponceau red staining as a normalizer (top), and graphs showing the individual variations between the resection margins and the tumors (down). Results are expressed as the mean ± SEM. RM: resection margin; T: tumor. Statistical test: paired Wilcoxon test (**, *p* < 0.01); *n* = 10. Molecular weight markers are indicated (in thousands) in the right margin.

Despite the unavailability of a specific antibody for investigating the expression of 67EBP on tissue sections, a previous study provided evidence that 67EBP can be identified using an anti-β-galactosidase antibody previously characterized to identify β-galactosidase and its variants including the 67EBP [27]. The expression of RPSA and 67EBP was, thus, evaluated by Western blotting analyses on tissue samples prepared from 10 patients (Series #1, Table 1), which included tumors of stages 2, 3, and 4 and their corresponding normal resection margins (Figure 2). Using Ponceau red staining as a normalizer, the expression of both RPSA and 67EBP in CRC tissues was found to be increased for the majority of the patients, while the overall β-gal expression was not significantly altered (Figure 2B). RPSA and 67EBP were, thus, significantly overexpressed in tumor tissues than in the resection margins, although no significant correlation was found between protein expression and the stage of the disease.

No significant correlation was observed between the expression of 67EBP and β-galactosidase in the tumor tissues (Figure S1).

**Figure 3:**
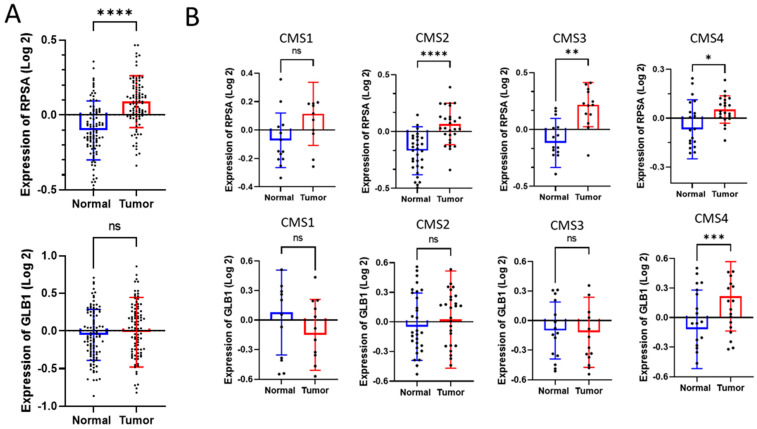
RPSA and GLB1 proteomic expression from CPTAC dataset. The proteomic expression levels of RPSA and GLB1 (β-galactosidase) were analyzed in 56 colorectal tumors (in red) and their matched normal mucosa samples (in blue) from the CPTAC dataset (Series #3, Table 1) and expressed as the logarithmic difference in protein abundance, measured by mass spectrometry. (**A**) Unshared log 2 ratio expression of RPSA (upper panel) and GLB1 (lower panel). (**B**) Expression of RPSA (upper panels) and GLB1 (lower panels) based on transcriptomic subtypes associated with CMS1 (microsatellite instability and immune infiltration), CMS2 (WNT and MYC signaling activation), CMS3 (KRAS mutation and metabolic deregulation), CMS4 (TGFβ activation in EMT and ECM remodeling). Data are expressed as the mean ± SEM. Statistical test: paired Wilcoxon test (ns, not significant; *, *p* < 0.05; **, *p* < 0.01; ***, *p* < 0.001; ****, *p* < 0.0001).

We further investigated the significance of RPSA and 67EBP in CRC using proteomic expression data from the Clinical Proteomic Tumor Analysis Consortium (CPTAC) for 104 patients, with clinicopathological characteristics depicted in Table 1 (Series #3). In this dataset, besides RPSA, only β-galactosidase (GLB1) was included since the 67EBP isoform was not distinguishable from GLB1. An increase in the RPSA expression in CRC tissues was noted compared with that in normal mucosa, unlike GLB1, which showed no significant difference (Figure 3A). A reanalysis considering the classification of CRC according to consensus molecular subtypes (CMSs) revealed interesting features. The RPSA expression was significantly increased in CRC tissues compared with that in normal colorectal tissues for the subtypes CMS2, CMS3, and CMS4, while the β-galactosidase expression was increased for the CMS4 subtype (Figure 3B). Moreover, expression analysis compared to the integrated phenotype of the tumor showed a significant increase in RPSA expression in epithelial, EMT (epithelium–mesenchyme transition), and hypermutated subtypes compared with a significant increase in β-galactosidase expression only in the EMT subtype (Figure S2A). However, a relative expression analysis of RPSA and β-galactosidase in CRC tissues compared to normal tissues by TNM stage showed no significant variation for both proteins across all stages (Figure S2B).

### 2.2. RPSA and 67EBP Transcript Expression in CRC Tissues

**Figure 4:**
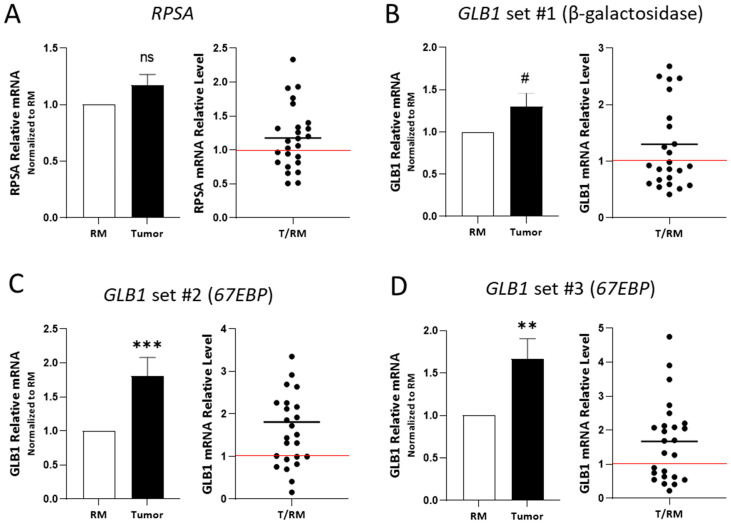
Expression of RPSA, GLB1, and GLB1 variant 67EBP transcripts in CRC tissues. Expression levels of *RPSA*, *GLB1,* and *GLB1* variant *67EBP* were evaluated at the transcript levels in 25 CRC samples and corresponding resection margins from the biobank collection (Series #2, Table 1) using quantitative RT-PCR. (**A**) Expression of *RPSA*. (**B**) Expression of *GLB1* using primer set #1 targeting both β-galactosidase and *67EBP* mRNA. (**C**) Expression of *GLB1* using primer set #2 targeting exons 2–5 specific to the mRNA splice variant of *67EBP*. (**D**) Expression of *GLB1* using primer set #3 targeting exons 5–7 specific to the mRNA splice variant of *67EBP*. *RPLP0* was used as the normalizer. (**A**–**D**, left): Expression levels were quantified using the Pfaffl method and compared to the resection margin. Results are presented as mean ± SEM. Statistical test: paired Wilcoxon test; (ns, not significant; #, *p* = 0.07; **, *p* < 0.01; ***, *p* < 0.001; (**A**–**D**, right): Graphs displaying the relative amounts of RPSA and 67EBP in individual tumor tissue (T) compared to the resection margin (RM). Red lines are for a ratio of 1.0. Sample size: *n* = 25.

The expression of *RPSA* and *67EBP* was also evaluated at the transcript level using RT-PCR on tissues from 25 patients (Series #2, Table 1), including tumors at all stages and their corresponding normal resection margins (Figure 4). The transcript levels of *RPSA* were not significantly altered in CRC tissues as compared with those in the resection margins when using primer set #1 (Table S1) with *RPLP0* as the reference gene (Figure 4A). This finding was confirmed using primer sets #1-3 using *B2M* as the reference gene (Figure S3). The mRNA levels of *GLB1*, using the primer set #1 targeting β-galactosidase mRNA (see Section 4 for details, Figure S4), showed a *p* = 0.07 significance between the CRC tissues and the resection margins (Figure 4B). Using primers targeting the specific spliced variant of *GLB1* encoding *67EBP* (see Section 4, Figure S4, primer sets #2 and #3), a significant increase in the transcript level was observed in CRC tissues compared with that in the resection margins (Figure 4C,D). However, no significant differences in the mRNA expression of *RPSA* and *GLB1* (β-galactosidase and *67EBP*) were observed when correlated with the CRC stage (Figure S5).

**Figure 5:**
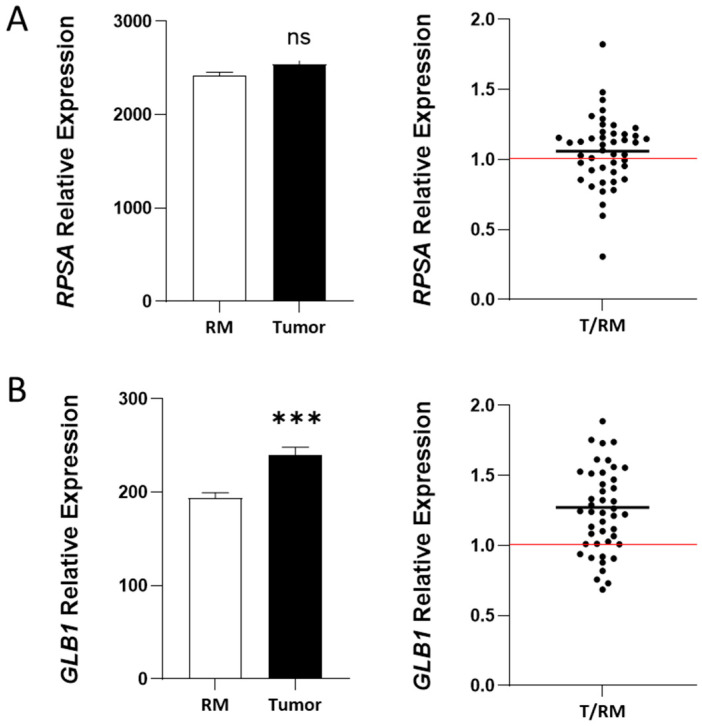
Expression of *RPSA* and *GLB1* transcripts using a GEO DNA microarray dataset from patients with colorectal cancer. Analysis of *RPSA* and *GLB1* transcript levels in primary tumors and their resection margins from the GEO microarray dataset GSE41258 (Series #4, Table 1) using the Affymetrix Human Genome U133A Array. Only pairs whose expression was available in both the primary tumor and the corresponding resection margin were used. (**A**) RPSA expression analyzed using probe 213801_x_at. (**B**) GLB1 expression analyzed using probe 201576_s_at. Results are presented as mean ± SEM. Statistical significance was determined using the paired Wilcoxon test (ns, non-significant; ***, *p* < 0.001). Red lines are for a ratio of 1.0. Sample size: *n* = 44.

We also obtained gene expression microarray data from publicly available human CRC datasets from the Gene Expression Omnibus (GEO) database GSE41258. The *RPSA* and *GLB1* expressions were analyzed using the data from 43 patients for whom information was available for tumors and corresponding resection margins. The clinicopathological characteristics of these datasets are depicted in Table 1 (Series #4). We found that *RPSA* was not modulated in tumor tissues relative to the resection margins (Figure 5A). In contrast, GLB1 showed increased expression in primary tumor tissues compared with the resection margins (Figure 5B). Although information for the *67EBP* variant was not available, it was available for *NEU1* and *CSTA*, the genes encoding the two other proteins implicated in the formation of a functional elastin-binding complex with 67EBP. The expressions of *NEU1* and *CSTA* were significantly increased in tumor tissues compared with those in the resection margins (Figure S6A,B). Levels of the *ELN* transcript (encoding elastin) were also increased in the CRC tissues (Figure S6C). No significant differences were observed in the transcript levels of *RPSA* and *GLB1* when correlated with the CRC stage (Figure S6D).

## 3. Discussion

The interaction between cancer cells and basement membrane components is crucial in colorectal carcinogenesis. Our findings indicate that RPSA-containing laminin receptor (RCLR) and 67EBP, which act as distinct receptors for laminin and/or elastin and shown recently to be the subject of confusion surrounding the molecular identity of 67LR [27], are both overexpressed in CRC.

The overexpression of 67LR has been previously reported by immunohistochemistry in ovarian, breast, thyroid, lung, gastric, and colonic malignancies using a variety of antibodies [18,19,28,29,30,31]. As shown above, the difficulty in interpreting these observations was the fact that some of these antibodies were directed toward a 67 kDa protein [18,19,28,29,32], likely corresponding to 67EBP as suggested in our previous work [27], or against the 37 kDa RPSA sequence [30,33], which shows immune cross-reactivity with 67EBP [27]. Incidentally, previous studies with intestinal cell lines revealed that RPSA was present in both normal and CRC cell lines [27]. RPSA was noted in normal intestinal crypt cells consistent with the pro-proliferative influence of this laminin receptor in these cells as well as the direct interaction of these cells with the YIGSR peptide [8]. In contrast, the 67EBP protein expression was below detection levels in normal HIEC-6 cells while present in CRC cell lines in agreement with its previously demonstrated expression in some malignancies [34,35]. qPCR analyses confirmed the expression of *67EBP* in colorectal cancer cells and its absence in normal intestinal crypt cells, although *GLB1*, from which the *67EBP* is derived by alternative splicing [36], was detected.

In tissue samples, an upregulation of both RPSA and 67EBP was observed by immunofluorescence using an anti-RPSA antibody that cross-reacts with 67EBP [27]. Western blot and qPCR analyses of CRC lysates were used to effectively discriminate between RPSA, 67EBP, and GLB1, which encodes β-galactosidase. The RPSA expression was found to be increased in CRC as assessed by Western blot as well as by CPTAC expression dataset analysis. Although it was previously suggested that the RPSA transcript levels were increased in CRC [37], qPCR analysis using three sets of primers and two normalization methods (RPLP0 and B2M) did not show a significant increase in the RPSA transcript levels in colorectal tumors, consistent with the microarray dataset analysis. This discrepancy between the protein and transcript expression levels of RPSA was similar to what was observed in CRC cells, suggesting a deregulation of the RPSA translation process in vivo. Incidentally, deregulation of the translation process has been shown to facilitate CRC progression and liver metastasis formation [38], while several mutations and signaling events implicated in the progression of CRC have been shown to contribute to translation deregulation. For instance, mutation of the APC gene has been shown to enable the transcription of genes involved in protein synthesis and the cellular stress response, leading to translation deregulation and tumor growth [39,40]. Alterations in the RAS/MAPK and PI3K/AKT signaling pathways in late stages of CRC appear to trigger the overexpression of eIF-type proteins, which regulate translation and ribosome biosynthesis [41,42]. Moreover, the oncogene MYC, which is involved in CRC cell–matrix interactions [43], has been shown to promote ribosome biosynthesis [44] and transcription of several translation initiation factors [45]. Increased levels of several ribosomal proteins, including RPL15 and RPS24, also promote the proliferation and migration of CRC cells [46,47,48]. In this context, it is noteworthy that expression analysis based on CPTAC datasets indicated that RPSA was increased in all integrated phenotype groups, suggesting that some mutations associated with colorectal carcinogenesis may impact RPSA translation. Furthermore, it has been proposed that microRNAs such as hsa-miR-16-5p, hsa-miR-615-3p, and hsa-miR-484 may interact with the RPSA mRNA [49]. Interestingly, although microRNAs can regulate the RPSA transcript by degrading it, certain microRNAs, such as miR-10a, have also been shown to positively regulate the expression of specific ribosomal proteins by binding the 5′UTR of their transcripts, thus controlling translation without affecting the transcript levels. These findings suggest that a similar mechanism could also involve the regulation of RPSA expression [50].

For 67EBP, an increase in the 67 kDa immunoreactive protein detected with the antibody against β-galactosidase was noted in 9 of the 10 CRC paired samples. This increased expression of 67EBP is consistent with previous findings of elevated 67EBP expression in fibrosarcoma [51] and lung cancer [52] and, in addition, aligns with the increased expression of a 67 kDa protein potentially misattributed to 67LR in various malignancies [25,53]. Furthermore, an increase in transcript levels was observed in tumors with primers specific to the *67EBP* splice variant, while primers targeting the *GLB1* mRNA showed only an increasing tendency (*p* = 0.07). At the protein level, β-galactosidase amounts were observed to be increased in only a subset of CRC samples, as previously observed in certain neoplasia such as prostate cancer [54]. Lysosomal exoglycosidases, including β-galactosidase and neuraminidase, have been shown to be increased in CRC [55], in which they may be involved in the degradation and remodeling of colonic neoplastic tissues [56]. However, the expression of β-galactosidase did not correlate with the 67EBP levels suggesting a distinct function and regulatory mechanism for the expression of these two proteins in colorectal carcinogenesis.

Expression analysis from the CPTAC and GEO microarray datasets did not allow us to distinguish between β-galactosidase and its variant corresponding to 67EBP. It is nevertheless noteworthy that an increase in β-galactosidase was observed at the protein level in tumors derived from the CMS4 transcriptomic subtype and the integrated phenotype corresponding to EMT as expected [57], although expression of the *GLB1* transcript was increased in CRC samples. While this information is limited relative to 67EBP itself, the expression of the two other proteins that contribute to forming a functional elastin-binding complex, *NEU1* and *CSTA*, as well as *ELN*, which encodes elastin, was found to be increased in CRC samples, suggesting a potential increase in the functional elastin-binding/secreting complex in CRC. In this context, it is interesting to note the pro-tumor properties of elastin [58], which, in CRC, could regulate adhesion, migration, and invasion [59].

Altogether, these results emphasize the potential impact of the overexpression of RCLR and 67EBP on CRC progression and their usefulness as biomarkers. However, this study has limitations, and future research is needed to confirm their specific roles and clinical utility. Indeed, on one hand, 67EBP is part of the elastin receptor complex, which has been reported to be involved in a variety of biological functions including the migration and invasion of normal and tumor cells [60] via its ability to interact with elastokines [61] and regulate TGFβ bioavailability [62]. More work is, thus, needed to further assess the implication of the elastin receptor complex in CRC. On the other hand, there are numerous cellular activities initially attributed to the 67LR, such as the modulation of cellular adhesion, migration, extracellular matrix remodeling, and apoptosis [24,63,64], as well as its ability to act as a receptor for viruses, bacteria, and prions [24,65] and to transduce the numerous green tea polyphenol EGCG effects [66], that need to be reassessed. However, prior to addressing these important issues as well as assessing the individual usefulness of these two receptors for CRC diagnosis, the identity of the RCLR will have to be elucidated while new tools targeting RCLR and 67EBP need to be developed. In this context, distinguishing RCLR from ribosomal RPSA and against the 67EBP variant of β-galactosidase using RNA-based or mass spectrometry approaches will be required. Indeed, although the hypothesis of a covalently bound partner to RPSA integrated at the membrane as a 67 kD laminin receptor has been excluded [27], the contribution of RPSA to a RCLR as a possible receptor complex needs to be better understood and its composition elucidated before exploring its biological functions relative to those attributed to the 67LR. In this context, it is interesting to note that the 67LR interaction with laminin, EGCG, and prions has been assigned to amino-acid sequences of RPSA [67,68,69], although the mechanism by which RPSA can reach the plasma membrane remains unknown. Among other limitations that require further work is the observed discrepancy between the RPSA protein and transcript levels. Indeed, further research is needed to evaluate post-transcriptional regulatory mechanisms, such as microRNA involvement, which could also provide novel diagnostic insights.

## 4. Materials and Methods

### 4.1. Human Colorectal Tissue Samples

The primary colorectal adenocarcinomas and their paired resection margin tissues were from patients undergoing surgical resection without prior neoadjuvant therapy. They were obtained from a previously described biobank [70]. The tissues were collected after the patients’ written informed consent, according to a protocol approved by the Institutional Human Subject Review Board of the Centre Hospitalier Universitaire de Sherbrooke. For protein extraction, 10 paired tissues (Series #1) were lysed in RIPA buffer (25 mM Tris–HCl pH 7.6, 150 mM NaCl, 1% NP-40, 1% sodium deoxycholate, 0.1% SDS, all from Sigma-Aldrich, Oakville, ON, Canada) and analyzed by Western blot for the detection of RPSA and 67EBP. For transcript analyses of the RPSA and GLB1 variants, quantitative RT-PCR was carried out on mRNA samples of 25 CRC tissues and their corresponding resection margins (Series #2). The tissues were processed, classified, and graded as previously described [70]. The clinicopathological parameters of the CRC patients and tumors for Series #1 and 2 are described in Table 1. This study was performed in accordance with a protocol approved by the Institutional Human Subject Review Board of the Centre Hospitalier Universitaire de Sherbrooke (#1991-17,90-18; 27 August 2024).

### 4.2. Expression Analysis Using Publicly Available Datasets of CRC

The gene expression analysis was carried out using the GSE41258 microarray dataset available in the Gene Expression Omnibus (GEO, https://www.ncbi.nlm.nih.gov/geo/, accessed on 5 June 2022) database (Series #3, Table 1). This series covering the expression profile of 390 samples from patients with CRC treated at Memorial Sloan–Kettering Cancer Center between 1992 and 2004 was carried out by DNA microarray, using the Affymetrix Human Genome U133A platform, on primary tumors, metastases, and normal mucosa [71]. The information available included the tumor stage. For expression analysis in tumors compared to normal mucosa, only pairs whose expression was available in both the primary tumor and the corresponding resection margin were used (n = 43). For proteomic expression analysis, we analyzed the proteomic data for tumors and resection margins from a cohort of CRC patients (Series #4, Table 1). The data were obtained from publicly available datasets of CPTAC CRC proteome data (http://www.linkedomics.org/admin.php, accessed on 5 June 2022) [72]. This series covered the expression profile of 56 patients with CRC. We analyzed the expression of RPSA and GLB1 in correlation with the transcriptomics subtypes; the expression of signature genes in cancer tissues related to epithelial, EMT, and hypermutated phenotypes; and the stage of the tumor.

### 4.3. Cell Lines

The colorectal cancer Caco-2/15 and SW480 (CCL-228) cells as well as the normal HIEC-6 (CRL-3266) cells were grown and tested as described previously [27]. Cells were routinely tested for the absence of mycoplasma contamination using the Myco Sensor PCR kit (Agilent, Mississauga, ON, Canada). All cell line identities were confirmed by short-tandem-repeat profiling cell authentication.

### 4.4. Immunofluorescence

For immunofluorescence on tissues, the preparation and optimum cutting temperature compound (Canemco-Marivac, Lakefield, QC, Canada) embedding of specimens and cryosectioning was performed as previously described [8] for RPSA immunolocalization. Nonspecific binding sites were blocked using 10% centrifuged fat-free milk in PBS containing 0.1% Triton for permeabilization for 1 h. The primary RPSA-antibody (Ab90073, Abcam, Cambridge, MA, USA) was incubated in blocking solution for 1 h at room temperature followed by secondary antibody incubation for 45 m at room temperature. 4′,6-Diamidino-2-phenylindole (DAPI) was used for nuclear staining. The negative control consisted of samples incubated without primary antibody. The slides were mounted using mounting medium (DAKO; Sigma-Aldrich, Oakville, ON, Canada) and observed with a Leica DM-RXA microscope (Leica Microsystems, Toronto, ON, Canada). The images were acquired using MetaMorph software v7.7 (Universal Imaging Corporation, West Chester, PA, USA). Stained tissues were also viewed with a Leica DM-RXA microscope. Images were acquired, and composites were generated with the MetaMorph Imaging System (Universal Imaging, West Chester, PA, USA).

### 4.5. RNA Extraction, Reverse Transcriptase, and Quantitative RT-PCR

The total RNA was extracted using RiboZol (VWR Life Science, Solon, OH, USA), and the extraction was performed according to the manufacturer’s instructions. The reverse transcription of mRNA was performed using the reverse transcriptase Omniscript (Qiagen, Germantown, MD, USA) as in [73]. Quantitative polymerase chain reaction (qPCR) assays were performed using Green-2 Go qPCR Low ROX Master Mix (BioBasic, Markham, ON, Canada) as previously described [73]. The primers used in this study for qPCR are described in Table S1. Gene expression was calculated according to the Pfaffl equation [74] using RPLPO or B2M as a validated normalizer [73]. For the *67EBP*, which is a variant of β-galactosidase generated by alternative splicing, we tested 3 sets of primers: set #1 from a common region of both the β-galactosidase transcript (*GLB1*) and *67EBP* and sets #2 and #3 specific for two distinct regions encoding *67EBP* (Figure S4A). Set #1 was found to amplify an expected fragment of 231 bp in HIEC-6, Caco-2/15, and SW480, while sets #2 and #3 amplified expected fragments of 98 and 200 bp, respectively, in Caco-2/15 and SW480 but not in HIEC-6 (Figure S4B).

### 4.6. Protein Extraction and Western Blot

Western blots were performed as previously described [8,9]. Briefly, cells were washed with phosphate-buffered saline (PBS), lysed using cell lysis buffer, and harvested in a 1× Laemmli buffer. The lysate was then heated to 95 °C, sonicated, and centrifuged at 15,000× *g* for 15 m. The supernatant containing the proteins was stored at −80 °C until use. The same amount of proteins was separated under denaturing and reducing conditions by 10–15% SDS-PAGE and transferred to nitrocellulose membranes (BioRad, Saint-Laurent, QC, Canada). Nonspecific binding sites were blocked using 5% fat-free milk (Blotto) in PBS-Tween 20 (0.1%, BioRad). The membranes were incubated overnight with the primary antibodies at 4 °C for 1 h at room temperature with the secondary antibodies coupled to HRP in the blocking solution. Positive bands were revealed using the enhanced chemiluminescence (ECL) kit (Immobilon, Millipore, Sigma-Aldrich, St. Louis, MO, USA), and the signal was detected using either a LI-COR Biosciences/Odyssey Imager reader and Image Studio Lite 5.2 software or via autoradiographic film (GE Healthcare, Mississauga, ON, Canada). Band quantification and densitometry analysis were performed using ImageJ v 1.53 software (National Institute of Health, Bethesda, MD, USA). Relative quantification was performed based on Ponceau red staining intensity or the expression of β-actin. The rabbit polyclonal primary antibodies used were anti-RPSA (Abcam, Ab90073, WB: 1/1000), anti-RPSA (Abcam, Ab246651, WB: 1/3000), anti-β-galactosidase (Abcam, Ab196858, WB:1/1000), anti-β-galactosidase (Boster, A01829-2, WB: 1/1000), and anti-α2 integrin (Abcam, Ab133557, WB:1/1000). A mouse anti-β-actin (Millipore, #MAB1501, WB:1/50,000) was used for normalization in some experiments. Secondary antibodies used for immunoblotting were anti-mouse #BA1050-05 and anti-rabbit #BA1054-05 HRP-linked ECL (Boster Biological Technology, Pleasanton, CA, USA; WB: 1:16,000).

### 4.7. Statistical Analysis

The data are expressed as mean ± SEM. Representative Western blots and immunostaining illustrations are shown. The normality of the data was previously validated using the Shapiro–Wilk test at a significance level of 0.05. Subsequently, the two-tailed Student *t*-test for unpaired samples, the paired Wilcoxon test, or an ANOVA analysis of variance followed by the Bonferroni multiple comparison test was used. Data presentation and statistical analysis including two-tailed Student’s *t*-test for unpaired samples were performed using Graph Pad Prism 10.1 (Graph Pad software; San Diego, CA, USA).

## 5. Conclusions

In conclusion, our study underscores the potential role of the two non-integrin laminin receptors RCLR and 67EBP in colorectal carcinogenesis, revealing their dual overexpression in CRC tissues. The increased RPSA protein expression suggests the involvement of RCLR in laminin binding and subsequent cellular proliferation and migration, despite the absence of significant changes at the transcript level, highlighting a potential deregulation in the translation process previously observed in CRC progression. Overexpression of 67EBP in CRC and its correlation with elastin binding and ECM remodeling emphasize its potential contribution to tumor progression, particularly within the EMT. Taken together, these findings, which revisit the historically puzzling molecular basis for the 67LR in CRC, provide new insights for a better understanding of the complex interactions between cells and basement membrane components in colon cancer progression. Future studies are, however, needed for optimizing specific detection methods and evaluating the prognostic value of RCLR and 67EBP expression in conjunction with other established biomarkers in CRC.

## Figures and Tables

**Table 1:** Clinicopathological parameters of the CRC patient series used in this work.

Series:	#1-Biobank	#2-Biobank	#3-CPTAC 3	#4-GSE41258
Use:	Protein expression	mRNA expression	Proteomics	Gene expression microarray
Total n:	10	25	104	43
Age:	73.6 ± 8.27	68.2 ± 13.3	65.3 ± 11.6	62.6 ± 15.9
Sex (F/M):	5/5	11/14	45/65	20/23
Stage TNM				
I	0	3	10	7
II	1	7	42	8
III	7	9	44	10
IV	2	6	8	18

## Data Availability

All the data are included within this paper.

## Supplementary Materials

The following supporting information can be downloaded at www.mdpi.com/article/10.3390/ijms26062564/s1.

## Abbreviations

The following abbreviations are used in this manuscript:

CRC	Colorectal cancer
67LR	67 kDa form of the laminin receptor (hypothetical)
67EBP	67 kDa elastin-binding protein
RPSA	Ribosomal protein SA
RCLR	RPSA-containing laminin receptor
BM	Basement membrane
β-gal	β-Galactosidase
CPTAC	Clinical Proteomic Tumor Analysis Consortium
CMSs	Consensus Molecular Subtypes
EMT	Epithelium–mesenchyme transition
TNM	Tumour, Node, Metastasis (Staging)
GEO	Gene Expression Omnibus
qPCR	Quantitative polymerase chain reaction
PBS	Phosphate-buffered saline
DAPI	4′,6-Diamidino-2-phenylindole
