# Supplementary material for: Expression of the RPSA-Containing and 67EBP Laminin Receptors in Relation to the Debatable Nature of the 67 kDa Laminin Receptor 67LR in Colorectal Cancer
Source: Int J Mol Sci. 2025 Mar 12;26(6):2564. doi: 10.3390/ijms26062564 (PMC11942345; doi:10.3390/ijms26062564)
Supplement: Supplementary file 1 [file ijms-26-02564-s001.zip › Supplementary Figure S1-S6.pdf]

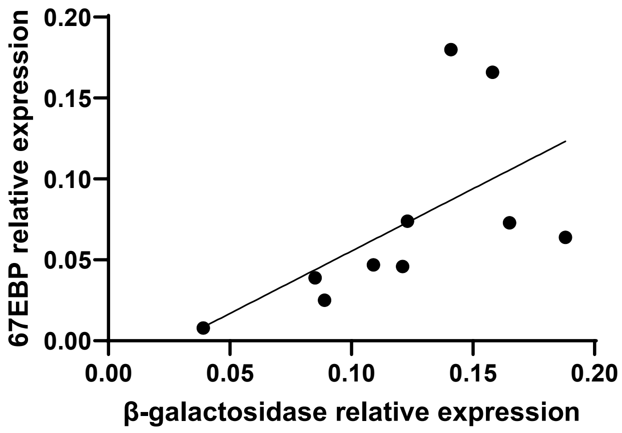

Figure S1. Expression of the β-galactosidase variant 67EBP was found to be distinct from that of the enzymatic β-galactosidase in CRC tissues. Graph showing the correlation analysis between the enzymatic β-galactosidase and 67EBP expression in CRC tissues; R square = 0.3524, not significant.

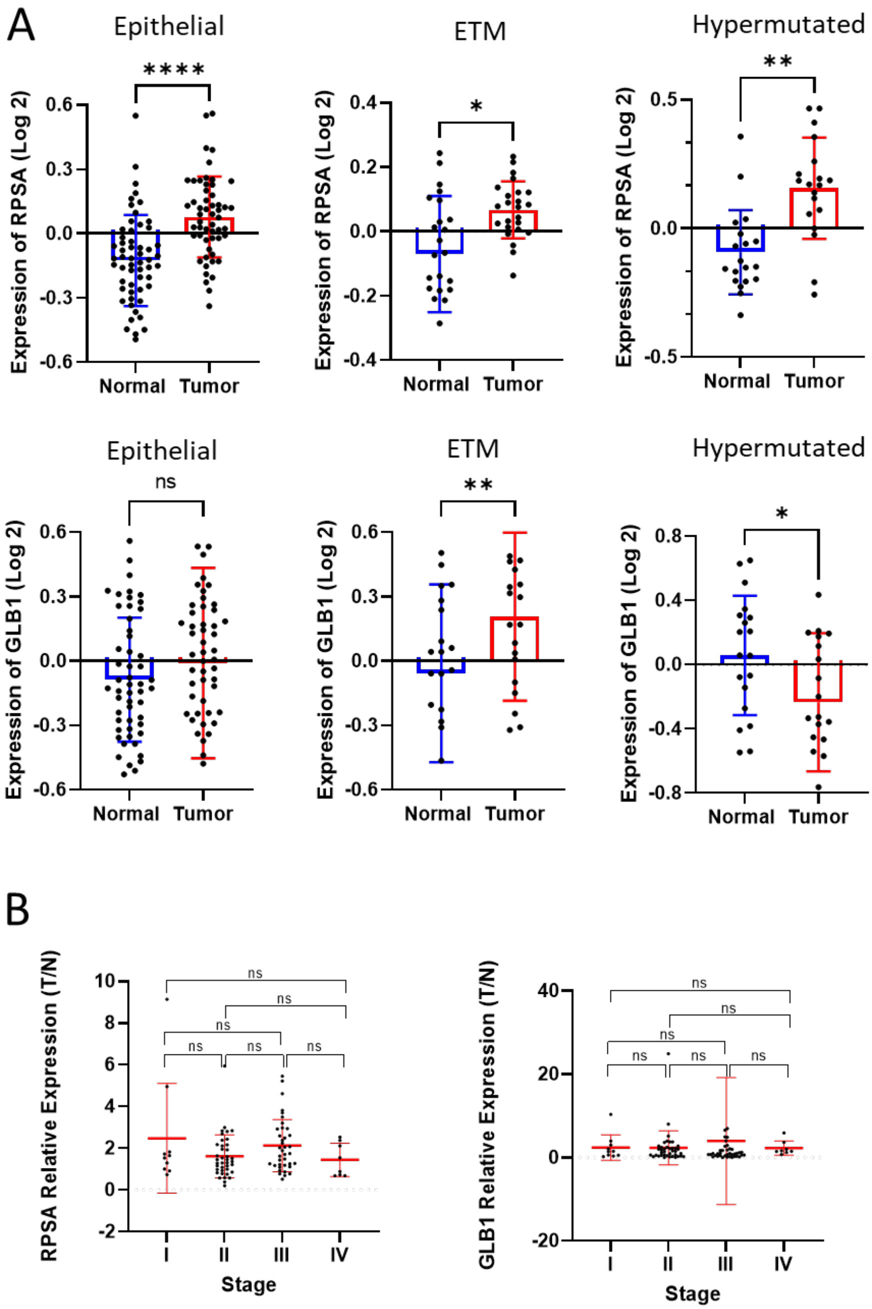

Figure S2. RPSA and GLB1 proteomics expression from CPTAC dataset. Proteomics expression of RPSA and GLB1 were analyzed in 104 colorectal tumor samples and their matched normal mucosa (dataset #3, Table 1). (A) Unshared log ratio expression of RPSA (upper panel) and GLB1 (lower panel) based on the integrated phenotype of the tumor. (B) Relative expression of RPSA and GLB1 in the tumor based on the stage of the disease. The data are expressed as the means ± SEM. Statistical test: paired Wilcoxon test. (ns, non-significant; *, p < 0.05; **, p < 0.01; ****, p < 0.001).

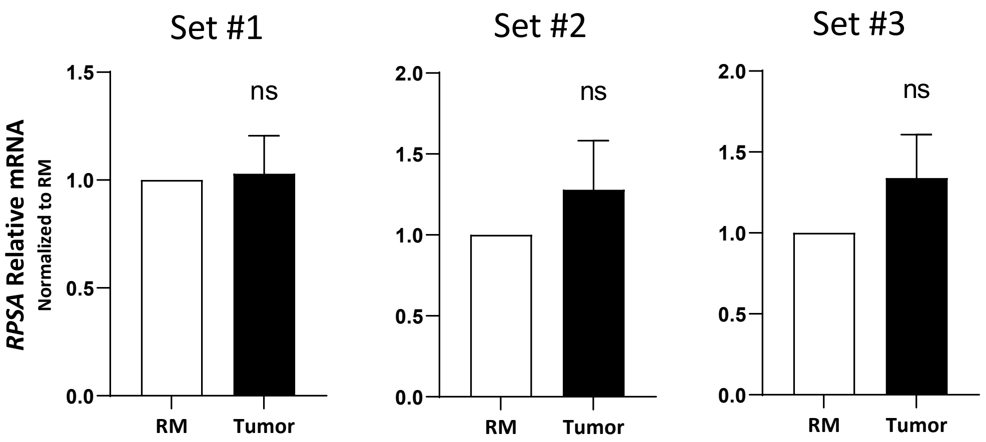

Figure S3. *RPSA* is not modulated at the transcript level in CRC tissues. Expression of *RPSA* was evaluated at the transcript level in CRC tissues and their corresponding resection margins from the Biobank sample collections (series #2, Table 1) using quantitative RT-PCR with three different sets of primers. No significant difference was observed in the expression levels. *B2M* was used as the reference gene, and expression levels were quantified using the Pfaffl method, normalized to the resection margins. Results are presented as mean ± SEM. Statistical analysis was performed using the paired Wilcoxon test, with no significant difference observed (ns); n = 25.

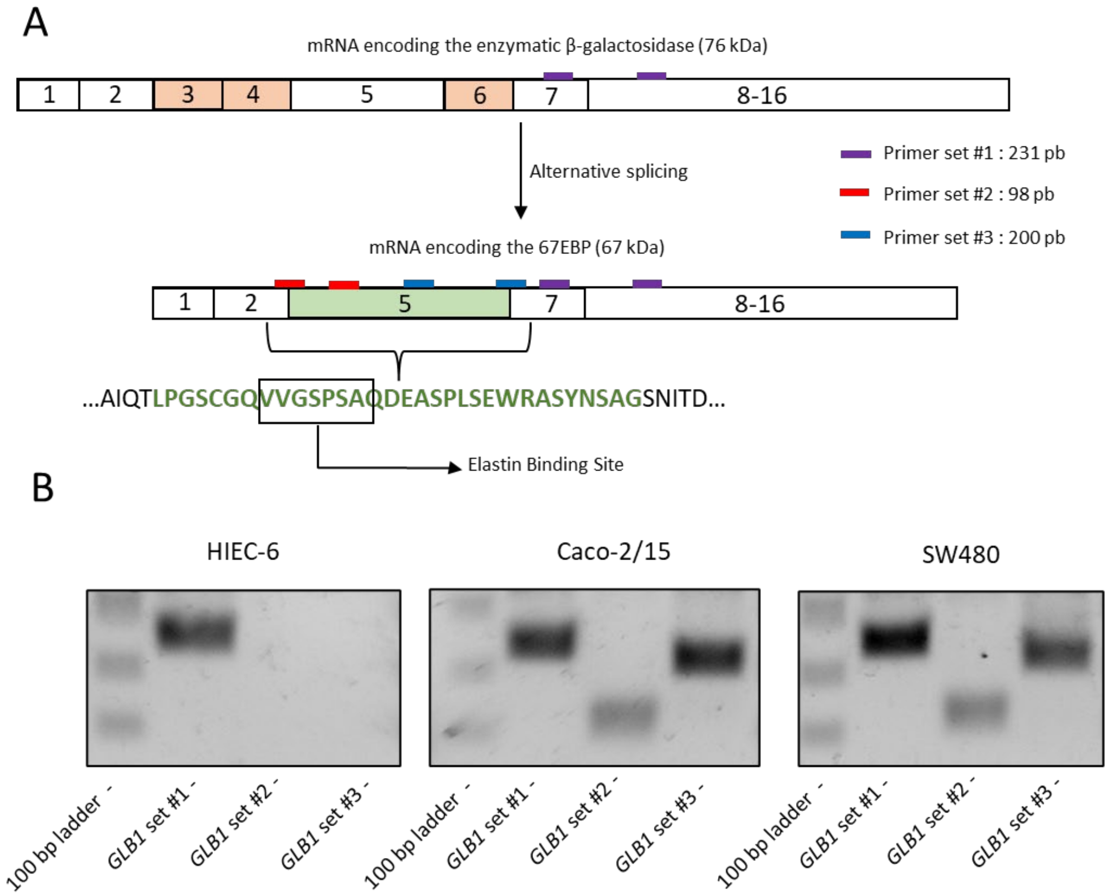

Figure S4. GLB1 and its splicing variants encoding 67EBP. Different sets of primers were used to target the GLB1 mRNA encoding β-galactosidase and its splice variant encoding the 67EBP. (A) Schematic representation of GLB1 mRNA and exons present in the spliced variant. Alternative splicing of the mRNA encoding enzymatic β-galactosidase (76 kDa) can produce a variant encoding an enzymatically inactive isoform (67 kDa) corresponding to the 67EBP. A frameshift caused by the deletion of exons 3 and 4 results in an altered exon 5 (green), leading to the emergence of an elastin binding site (framed sequence) on the 67EBP isoform. Primer set #1 targets the exons 7 and 8 on both mRNA, producing a 231 bp fragment. Primer set #2 overlaps exons 2-5, producing a 98 bp fragment, while primer set #3 overlaps exons 5-7, producing a 200 bp fragment. (B) Gel analysis of the PCR products to confirm the amplification of the expected fragment sizes from sub-confluent Caco-2/15 and SW480 CRC cells, with normal HIEC-6 cells used as a control. bp: base pairs.

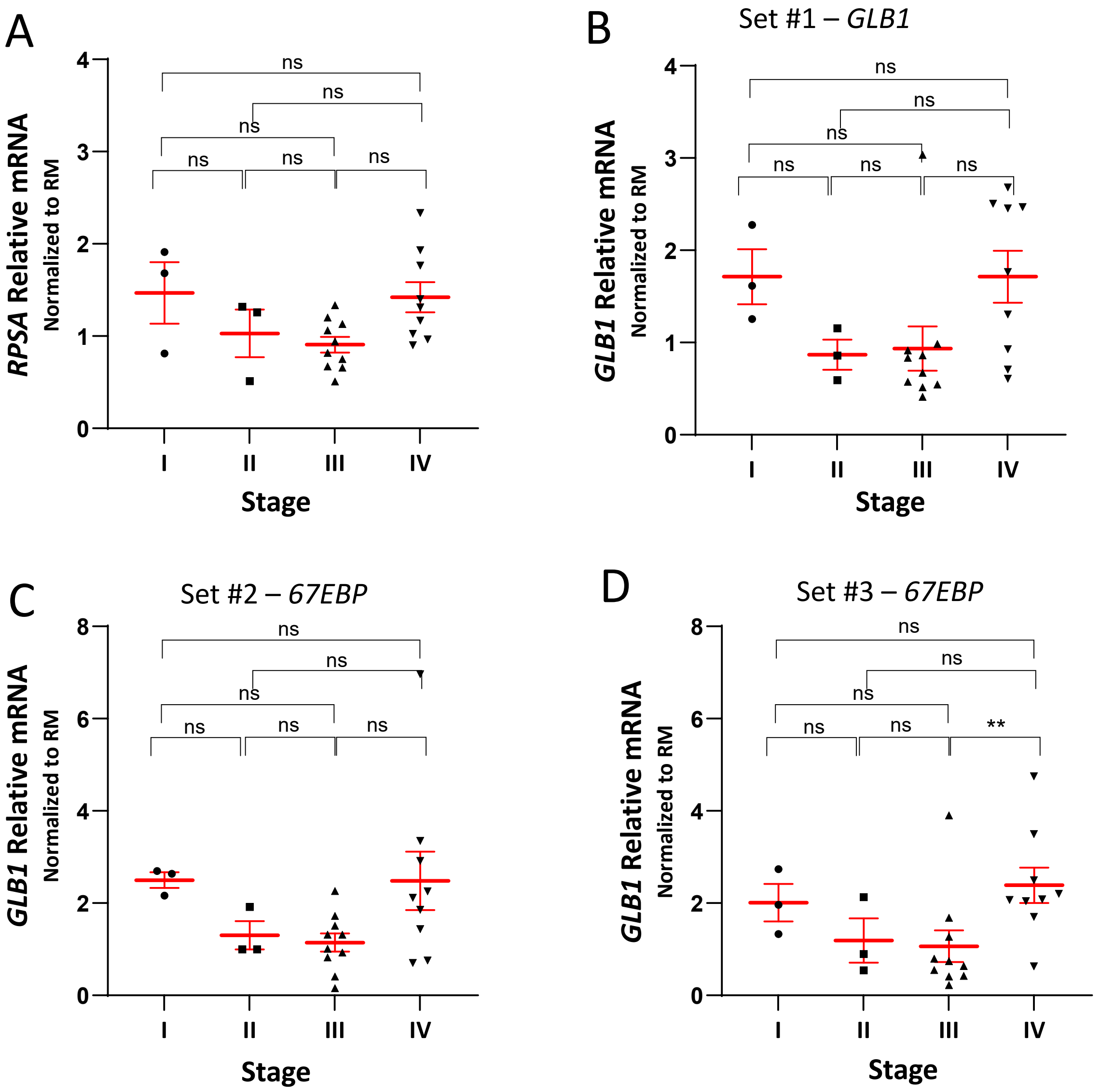

Figure S5. (A) *RPSA* and (B-D) *GLB* mRNA expression is not modulated in association with the stage of CRC. Expressions of *RPSA* and *GLB1* were evaluated at the transcript level in CRC tissues and their resection margins from the Biobank sample collection (series #2, Table 1) using quantitative RT-PCR. No significant difference was observed in the expression levels relative to the stage of the disease. *RPLP0* served as the reference gene, and expression levels were quantified using the Pfaffl method relative to the resection margin. Results are expressed as mean ± SEM. ns, non-significant, n = 25.

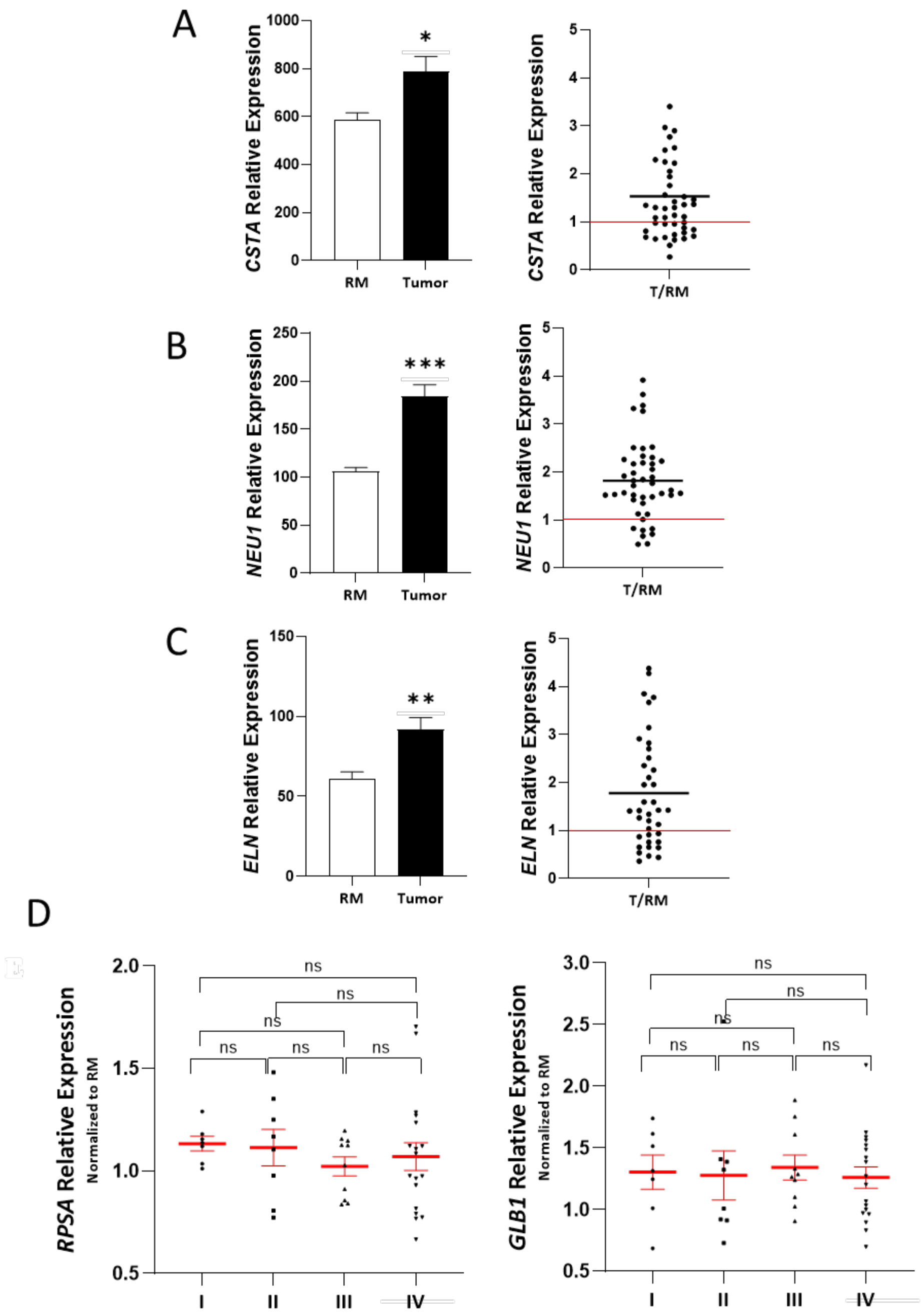

Figure S6. Expression of transcripts encoding elastin and its receptor in CRC samples. Analysis of transcript level expression in CRC tissues and their resection margins from the GEO microarray dataset GSE41258 (series #4, Table 1) using the Affymetrix Human Genome U133A Array. Transcript expression of (A) *CSTA* using probe 200661_at (B) *NEU1* using probe 208926_at (C) *ELN* using probe 212670_at (D) *RPSA* and *GLB1* transcript level in association with the stage of the disease. Expression levels were normalized to resection margins. Results are expressed as mean ± SEM. Statistical test: paired Wilcoxon test. (ns, non-significant; *, $p < 0.05$; **, $p < 0.01$; ***, $p < 0.001$); n = 43.
