# Supplementary material for: Expression of the RPSA-Containing and 67EBP Laminin Receptors in Relation to the Debatable Nature of the 67 kDa Laminin Receptor 67LR in Colorectal Cancer
Source: Int J Mol Sci. 2025 Mar 12;26(6):2564. doi: 10.3390/ijms26062564 (PMC11942345; doi:10.3390/ijms26062564)
Supplement: Supplementary file 1 [file ijms-26-02564-s001.zip › Table S1.pdf]

Table S1. Primers used for qPCR in this study

Gene symbol	Forward primer	Reverse primer
RPSA set #1	5’-CCAATATCCTCACGGACTGG-3’	5’-GGGCACAAACAAGGTCAACT-3’
RPSA set #2	5’-GAGCTCACTCAGTGGGTTTGATG-3’	5’-GCAGCAGCCTGCTCTTCTTTT-3’
RPSA set #3	5’-ATGTCCTGCAAATGAAGGAGG-3’	5’-TGGAAGTCAAGATTGGTGCCA-3’
GLB1 set #1	5’-ACTCTGGACCTTCTGGTAGAG-3’	5’-AACTCATCCAACTACACGCTC-3’
GLB1 set #2	5’-TGAACGCCATCCAGACATTAC-3’	5’-TATCAGAATGGAGGGCCAGT-3’
GLB1 set #3	5’-TAACAGTGCAGGCAGCAA-3’	5’-CGAGTGTGAACTTGTACATGT-3’
RPLP0	5'-GCAATGTTGCCAGTGTCTG-3'	5'-GCCTTGACCTTTTCAGCAA-3'
B2M	5’-GTGCTCGCGCTACTCTCTC-3’	5’-GTCAACTTCAATGTCGGAT-3’
